# HIV infection in Eastern and Southern Africa: Highest burden, largest challenges, greatest potential

**DOI:** 10.4102/sajhivmed.v22i1.1237

**Published:** 2021-05-28

**Authors:** Erica Parker, Melinda A. Judge, Eusebio Macete, Tacilta Nhampossa, Jienchi Dorward, Denise C. Langa, Caroline De Schacht, Aleny Couto, Paula Vaz, Marco Vitoria, Lucas Molfino, Rachel T. Idowu, Nilesh Bhatt, Denise Naniche, Peter N. Le Souëf

**Affiliations:** 1 Faculty of Health and Medical Sciences, The University of Western Australia, Perth, Australia; 2 Manhiça Health Research Centre, Manhiça, Mozambique; 3 Nuffield Department of Primary Care Health Sciences, University of Oxford, Oxford, United Kingdom; 4 Centre for the AIDS Programme of Research in South Africa (CAPRISA), University of KwaZulu-Natal, Durban, South Africa; 5 Department of Surveillance, Instituto Nacional de Saúde, Maputo, Mozambique; 6 Friends in Global Health, Maputo, Mozambique; 7 National STI, HIV/AIDS Programme, Ministry of Health, Maputo, Mozambique; 8 Fundaçao Ariel Glaser contra o SIDA pediátrico, Maputo, Mozambique; 9 Department of HIV/AIDS, World Health Organization, Geneva, Switzerland; 10 Médecins Sans Frontières, Maputo, Mozambique; 11 Center for Global Health, Centers for Disease Control and Prevention, Maputo, Mozambique; 12 Elizabeth Glaser Pediatric AIDS Foundation, Maputo, Mozambique; 13 Barcelona Institute for Global Health (ISGlobal), Spain

**Keywords:** HIV epidemiology, public health, risk factors, vulnerable populations, prevention and control, early diagnosis

## Abstract

**Background:**

The burden of HIV is especially concerning for Eastern and Southern Africa (ESA), as despite expansion of test-and-treat programmes, this region continues to experience significant challenges resulting from high rates of morbidity, mortality and new infections. Hard-won lessons from programmes on the ground in ESA should be shared.

**Objectives:**

This report summarises relevant evidence and regional experts’ recommendations regarding challenges specific to ESA.

**Method:**

This commentary includes an in-depth review of relevant literature, progress against global goals and consensus opinion from experts.

**Results:**

Recommendations include priorities for essential research (surveillance data collection, key and vulnerable population education and testing, in-country testing trials and evidence-based support services to improve retention in care) as well as research that can accelerate progress towards the prevention of new infections and achieving ambitious global goals in ESA.

**Conclusion:**

The elimination of HIV in ESA will require continued investment, commitment to evidence-based programmes and persistence. Local research is critical to ensuring that responses in ESA are targeted, efficient and evaluated.

## Introduction

In the decades since HIV-1 first emerged, the response has been marked by strong global commitments, extensive education campaigns and the development of improved tests and life-saving antiretroviral treatments (ART) that are reaching more and more individuals.^1^ With evidence-based prevention and treatment strategies available, nations have united to set goals, with the end of the HIV epidemic potentially attainable by 2030.^2^

One hallmark concept in the fight against HIV has been the ‘know your epidemic, know your response’ approach to deliver programmes in different settings.^3^ More than 70% of persons living with HIV (PLWH) reside in sub-Saharan Africa (SSA), where resources for healthcare are disproportionately limited. Eastern and Southern Africa (ESA), in particular, continues to record the highest rates of HIV-1 prevalence and incidence worldwide.^4^ In this region, knowing where and among whom the infection is spreading has been challenging, and key populations are only recently being highlighted.

A second hallmark of the fight against HIV has been the Joint United Nations Programme on HIV/AIDS (UNAIDS) ‘Fast-Track’ targets, whereby 90% of PLWH should know their status, 90% of those diagnosed should receive ART and 90% of those on ART should achieve viral suppression by 2020 (‘90-90-90’).^5^ Despite remarkable progress towards these targets in ESA, the sheer scale of the epidemic in this region leaves much to be done.^6^ In the next decade, efforts must be redoubled for raised targets of 95-95-95 by 2030.^7^ Programmes on the ground have identified region-specific challenges to be overcome and lessons that should be broadly shared with ESA and potentially with many communities globally.

An important barrier preventing progress in ESA is the timely detection and treatment of acute HIV infections.The earliest stage of HIV infection is characterised by high viral loads and a high potential for onward transmission, but it is typically missed using existing testing algorithms.^8^ As ART coverage improves, the proportion of transmissions attributable to undiagnosed acute HIV infection increases.^9^ Furthermore, new HIV infections disproportionately affect key populations.^10^ Affordable testing solutions for acute HIV detection in high-prevalence, resource-limited settings are needed.^9^

As the 2020 deadline passed, trends indicated that the 90-90-90 targets were not reached across most of ESA.^2^

Renewed efforts looking ahead to the 2030 UNAIDS targets of 95-95-95 will be required. To this end, a group of regional experts was invited to collate their expertise, with a view to addressing the local challenges that prevent the achievement of global goals.

## State of the global epidemic

**FIGURE 1:**
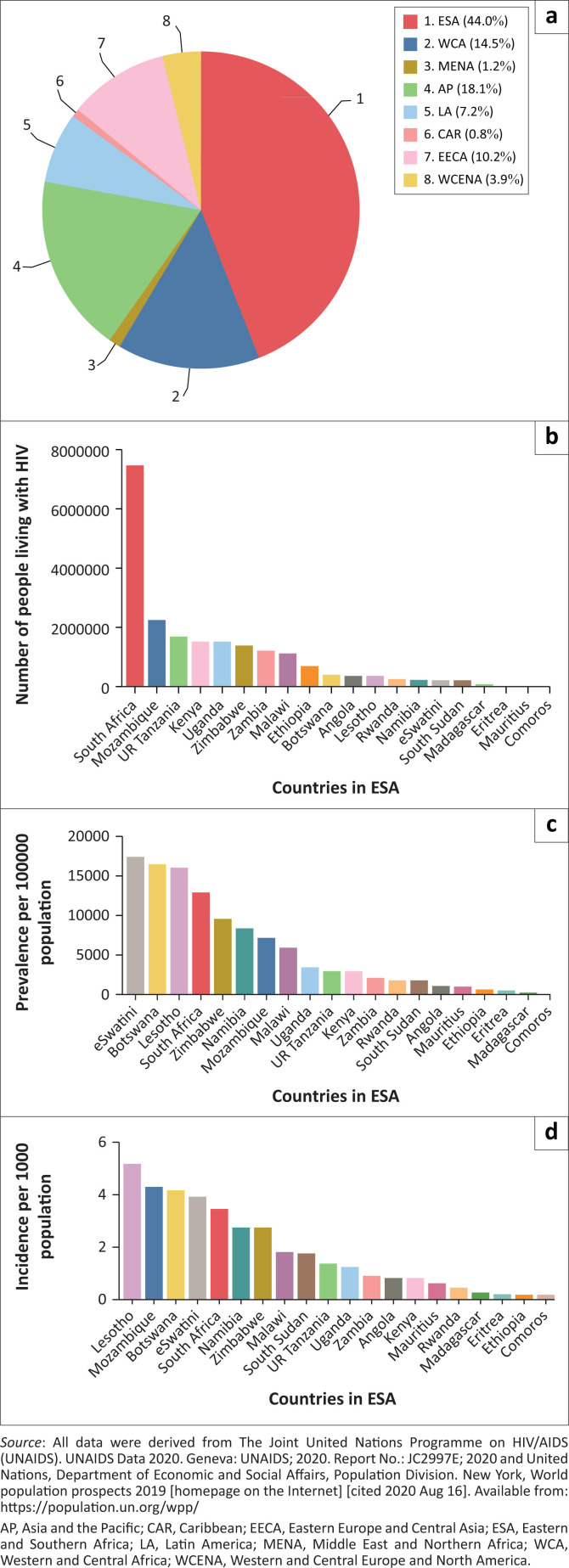
(a) Worldwide distribution of new HIV infections identified by UNAIDS in 2019. (b) Distribution of people living with HIV infection in ESA in 2019. (c) Prevalence of HIV infection per 100 000 population among countries in ESA. (d) Incidence of HIV infection per 1000 population among countries in ESA.

According to UNAIDS, there were an estimated 38 million PLWH worldwide at the end of 2019, with 1.7 million new infections and 690 000 AIDS-related deaths that year (Figure 1).^10^ A successful vaccine and functional cure for HIV are yet to be developed, and lifelong ART remains the cornerstone of management.

The 2020 UNAIDS report highlights a ‘prevention crisis’.^10^ Programmes aiming to prevent new HIV infections (such as education, barrier contraception, voluntary male medical circumcision and pre-exposure prophylaxis [PreP]) must be a priority alongside test-and-treat programmes and must appropriately target key populations and their partners, who comprise 62% of new HIV infections globally.^10^

## HIV epidemiology in ESA

**FIGURE 2:**
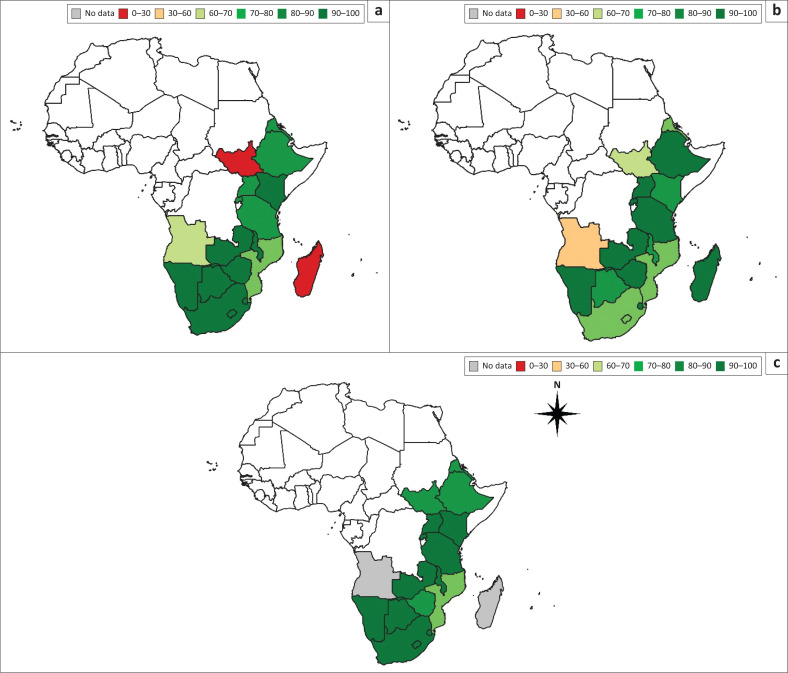
Data on the ESA 90-90-90 goals in all ages by country from the UNAIDS data 2020 report.^10^ (a) First 90; (b) second 90; (c) third 90.

Regional HIV epidemics look markedly different across the world and require tailored responses. In ESA, there are 20.7 million adults and children living with HIV (54% of global HIV prevalence),^10^ and in 2019 44% of all new infections occurred here.^10^ Key populations make up an estimated 28% of new infections in ESA.^10^ In some areas, reasonable progress has been made towards the Fast-Track targets (e.g. eSwatini, Namibia and Zambia); in other areas, progress is more limited (e.g. Mauritius and South Sudan). An estimated 87% of PLWH in ESA are aware of their status (the ‘first 90’; Figure 2); however, this figure ranges from 15% to 98% between countries.^10^

Of those diagnosed with HIV in ESA, approximately 83% have commenced ART (the ‘second 90’).^10^ This figure ranges from 37% to 98% between countries,^10^ highlighting deep inequities within and across countries. Of those on treatment, 90% have achieved viral suppression (the ‘third 90’); this figure ranges from 68% to 97% between countries, with 2 of 21 countries unable to provide estimates. Combatting the epidemic in ESA is a multifaceted challenge, and progress must occur within a broader context of socio-economic development. Despite some successes, the 2020 milestones were not achieved in many countries across ESA, and the greatest challenges persist as the focus shifts to achieving the new 95-95-95 targets.

## Challenges for achieving 95-95-95 in ESA

### The first 95

In high HIV prevalence settings, obtaining accurate measures of the first 95 is challenging. In ESA, HIV care commonly takes place in rural settings, utilising paper-based records.^11^ To estimate the first 95, the denominator is typically the number of people testing positive for HIV during randomised household or community-based serosurveys and/or at antenatal clinics; the numerator is those among them known to have previous positive results (either disclosed to surveyors or identified in medical records).^12^

Rates of non-disclosure can be high.^13^ When people are retested in southern Mozambique, non-disclosure of previous results occurs in over one-third of people, but the rate is higher for tests performed in a community setting (38.9%) or initiated by the provider (29.4%) than in those presenting for voluntary testing (13%).^13^ Similar findings have been described in Tanzania and Malawi.^14,15^ Cross-checking survey responses against medical records is impossible in many countries where HIV testing is performed anonymously.^11^ The high percentage of HIV-positive people who do not disclose, and are thus repeatedly deemed recently infected, leads to an overestimation of new HIV cases and an underestimation of progress towards the first 95. In Mozambique, non-disclosure resulted in underestimation of the first 95 by around 8.5%.^13^

Improving testing coverage to achieve the first 95 is feasible but must be accompanied by interventions that support the whole care cascade. In the Treatment as Prevention (TasP) trial of universal test-and-treat in KwaZulu-Natal, South Africa, repeated rounds of home-based testing increased the proportion of people knowing their HIV status to 91.5%.^16^ However, only 58.0% of these individuals commenced ART; many did not link to care.^16^ This suggests that to reach 95-95-95, all three targets must be understood and addressed in parallel.^17^

### The second 95

The second 95 is more readily measured, as countries (or their health facilities and non-governmental organisations) generally have stronger records on the number of people receiving ART. According to World Health Organization recommendations, early ART commencement has reduced HIV/AIDS-related mortality, with some models showing an estimated 75% fewer deaths per annum.^18^ Test-and-treat strategies recommending commencement of ART within 14 days of a positive diagnosis (independent of the CD4+ T-cell count) are relatively recent in most of SSA,^19^ and uptake has been commendable.^20^ Broader implementation is limited not only by the political will but also by the resources required to upskill staff and provide a sustainable treatment supply. Given the scale of the epidemic in ESA, the rollout of any advances in treatment regimens to the front lines can present a formidable challenge. The system’s fragility has been highlighted by COVID-19 over the past year, with reports of delays in the delivery of treatment stock from international suppliers, depleted national stockpiles and periods of lockdown limiting individuals’ access to HIV medications.^21^

Based on country guidelines, in 2014–2015, of those eligible for ART in Manhiça, Mozambique, 83.7% started ART within 3 months.^22^ In July 2016, Mozambique phased in the implementation of test-and-treat and undertook qualitative research into the patterns of ART initiation or refusal.^23,24^ The acceptance of treatment depends on the availability and accessibility of services, as well as appropriate and considered explanations following diagnosis.^25^

Linkage to care is improved by the desire to live, family support and subjective illness.^23,24^ Barriers to linkage to care include the fear of dissemination of one’s HIV status, feeling subjectively healthy, migration, health system issues and fears of discrimination.^23,24,26^

### The third 95

Achieving viral suppression requires retention in care, maintenance of ART and regular testing of the HIV viral load. Retention in care is improved by feeling better after ART initiation, confidence in the health system and support from family and providers.^27^ Communication about continuing treatment despite feeling better also helps.^24^ Barriers to retention include provider authoritarianism, which limits patient autonomy and engagement in their healthcare, and the adverse effects of ART.^27^ Men across the region are a hard-to-reach group; they test less, and more abandon ART after initiation.^22^ There are additional complexities related to paediatric care, such as the health literacy of parents and their confidence in managing HIV. For children, retention is highest when both mother and child register concurrently.^28^ Innovative strategies to improve testing uptake and support early ART initiation and nutritional supplementation can improve retention.^29,30^

Before the introduction of the universal test-and-treat programme, there were concerns that commencing ART among people with high CD4+ T-cell counts would overburden the health system and that those feeling healthy would not adhere to treatment.^31^ However, of the PLWH in KwaZulu-Natal with CD4+ T-cell counts of > 500 cells/µL, 78% accepted ART,^32^ 86% were adherent^33^ and 96% achieved viral suppression.^34^ Furthermore, retention in care and viral suppression were similar among people who initiated ART with CD4+ T-cell counts of > 500 cells/µL compared to those with lower CD4+ T-cell counts.^35^

Measuring the third 95 requires country-wide laboratory systems capable of processing large volumes of viral load requests and returning results; thus, many ESA countries score poorly.^10^ For example, in Mozambique, only 45% of PLWH achieve documented viral suppression^10^; however, viral load testing is available to few, particularly in rural settings.^36,37^ Under-developed laboratory systems also delay diagnoses of virological failure, leading to increased transmission, illness progression and treatment resistance.^38^ Point-of-care (POC) viral load testing improved viral suppression, retention in care and the communication of results to patients in KwaZulu-Natal,^39^ and it proved feasible and cost-effective in Botswana and Zambia.^40,41^ Further development of centralised, high-throughput laboratory-based testing alongside decentralised POC testing will be crucial to ensure adequate monitoring of viral suppression throughout ESA.

## The impending challenge of acute HIV infections

Acute HIV infection is commonly defined as the period prior to seroconversion, between 3 and 12 weeks in duration.^42,43^ Gene expression is vastly upregulated in the initial months, driving inflammation, immune responses and cell turnover.^44^ This correlates with a substantial peak in viral load, meaning the risk of onward transmission during acute HIV is 8–25 times higher than during chronic infection.^45,46,47^ The estimated prevalence of acute HIV infection in ESA is 1% – 3%.^48,49,50,51^ Undiagnosed acute HIV is particularly concerning for the following: pregnant and breastfeeding women who have poorer health outcomes as well as increased perinatal transmission risk^52,53,54,55^; people who received blood transfusions screened for HIV serology but not viral load^56,57^; and those who started PreP when already infected, as this may confer an increased risk of drug resistance mutations.^58^

The earliest time period that an acute HIV infection can be detected is 5–14 days by nucleic acid amplification.^59^ This is not feasible in low-resource settings, so other options include viral load POC testing (Gene Xpert^60^ and AlereQ^61^), p24 antigen testing (if developed into rapid tests),^62^ non-viral immune response biomarkers (e.g. IP-10)^63^ or a symptom/risk score.^8,64^ Rapid testing and ART for all HIV-seropositive individuals remains the priority; however, a focus on this alone will miss seronegative HIV-infected individuals. As ART coverage increases, the proportion of HIV transmission attributable to acute HIV will increase. Affordable rapid tests for p24 or non-viral immune markers combined with a risk score may be the best way to identify acutely infected individuals in high-HIV-burden, low-resource settings.

## Disproportionate impact of new HIV infections on key and vulnerable populations

Of the 1.7 million new HIV infections in 2019, 62% occurred in key populations and their sexual partners.^6^ Key populations include men who have sex with men,^65^ people who inject drugs,^66^ female sex workers^67^ and transgender people.^6^ Vulnerable populations at increased HIV risk in ESA include prisoners,^6,68^ long-haul truck drivers,^69^ mobile mining workers,^70^ migrants^71^ and serodiscordant couples.^6^ Also at disproportionately high risk of HIV infection are young women,^72^ who are 2–3 times^73,74^ more likely to be newly infected than their 15–24-year-old male counterparts.

Pregnant and breastfeeding women and their infants are an often-overlooked vulnerable population.^52^ Infants of mothers who acquired HIV during pregnancy or postpartum are at increased risk of HIV transmission compared to infants of chronically HIV-infected mothers.^52^

Approximately 45% of new global infections in 2019 were in ESA.^6,10^ No country in ESA has sufficient data to describe the size of their key populations,^10,75^ although several have commenced population-specific mapping (Table 1).^76^ Control of HIV in these populations will contribute to the deceleration of the HIV epidemic in the general population. National surveys of key populations biennially are recommended, as knowing the epidemic is the first key to design the response.

**TABLE 1:** Prevalence of HIV among certain key and vulnerable populations in ESA.

Country	HIV prevalence among				Reference
	MSM (%)	Sex workers (%)	PWID (%)	Prisoners (%)	
Angola	2.0 [2017]	8.0 [2017]	-	15.9 [2017]	^10^
Botswana	14.8 [2018]	42.2 [2018]	-	-	^75^
Comoros	0.0 [2018]	0.3 [2017]	1.8 [2017]	-	^10^
Eritrea	-	10.4 [2014]	-	1.4 [2019]	^10, 75^
eSwatini	12.6 [2015]	60.5 [2015]	-	34.9 [2015]	^75^
Ethiopia	-	24.3 [2014]	6 [2018]	4.2 [2016]	^75, 77, 78^
Kenya	18.2 [2011]	29.3 [2011]	18.3 [2011]	5.7 [2016]	^78, 79^
Lesotho	32.9 [2014]	71.9 [2014]	-	31.4 [2017]	^10, 75^
Madagascar	14.9 [2014]	5.5 [2016]	8.5 [2016]	0.3 [2018]	^75^
Malawi	6.8 [2019]	55.0 [2018]	-	19.0 [2019]	^10^
Mauritius	17.2 [2015]	15.0 [2015]	32.3 [2017]	17.3 [2017]	^75^
Mozambique	3.1–9.1 [2015]	17.8–31.2 [2016]	19.9–50.1 [2019]	24.0 [2019]	^75, 80, 81, 82^
Namibia	12.4 [2009]	40.7 [2016]	-	-	^75, 83^
Rwanda	4.0 [2016]	45.8 [2016]	-	-	^75^
Seychelles	13.2 [2013]	4.6 [2015]	23.0 [2019]	9.9 [2019]	^10, 79^
South Africa	18.1 [2018]	57.7 [2015]	21.8 [2018]	11.1 [2019]	^75, 79^
South Sudan	-	11.4 [2019]	-	5.3 [2016]	^10, 84^
Uganda	13.2 [2013]	31.3 [2017]	17.0 [2017]	4.0 [2019]	^10, 79^
UR Tanzania	8.4 [2018]	15.4 [2018]	15.5 [2013]	6.7 [2015]	^10, 79^
Zambia	-	48.8 [2017]	-	27.4 [2015]	^10^
Zimbabwe	21.1 [2019]	42.2 [2019]	-	28.0 [2015]	^10^

Note: Data on the HIV prevalence among transgender people are not presented in this table because of a lack of data on this key population in ESA; of the vulnerable populations, only prisoners and incarcerated people have sufficient data in ESA to be presented in this table.

MSM, men who have sex with men; PWID, people who inject drugs; [year], year of publication.

## Recommendations for research priorities

- Promote and expand local prevention research, including programme and policy evaluations.
- Investigate and implement methods to improve the accessibility of HIV education and testing, including routine surveillance, particularly for key populations.
- Support in-country trials of viral load and CD4 T-cell count POC testing and the surrounding services required to improve ART adherence, clinical management and retention in care.

## Conclusion

The road to HIV elimination in ESA requires continued strong and sustained national and international investment, commitment to evidence-based programmes and persistence. The region contains over half of the world’s population of PLWH and continues to have major challenges to achieving 90-90-90, let alone the looming target of 95-95-95. The priority must remain diagnosing, treating and virally suppressing all existing HIV infections. However, in high-prevalence settings, the prevention of new infections and early diagnosis of acute infections remain important goals. Research must ensure that responses in the region are targeted, efficient and evaluated. In particular, ESA will benefit from strengthened surveillance and key and vulnerable population research, in-country development and validation of HIV tests, and supported rapid transition to new ART regimens to ensure sustainable progress towards important global goals.
